# Categorical and Dimensional Approaches to Examining the Joint Effect of Autism and Schizotypal Personality Disorder on Sustained Attention

**DOI:** 10.3389/fpsyt.2020.00798

**Published:** 2020-08-07

**Authors:** Ahmad Abu-Akel, Ruth C. M. Philip, Stephen M. Lawrie, Eve C. Johnstone, Andrew C. Stanfield

**Affiliations:** ^1^ Institute of Psychology, University of Lausanne, Lausanne, Switzerland; ^2^ Tailor Ed Foundation, Edinburgh, United Kingdom; ^3^ Division of Psychiatry, University of Edinburgh, Edinburgh, United Kingdom; ^4^ Patrick Wild Centre, University of Edinburgh, Edinburgh, United Kingdom

**Keywords:** attention, comorbidity, executive function, inhibition, schizotypy, The Sustained Attention Response to Task (SART), vigilance

## Abstract

**Introduction:**

Accumulating evidence for the co-occurrence autism spectrum disorder (ASD) and schizotypal personality disorder (SPD) at both the diagnostic and symptom levels raises important questions about the nature of their association and the effect of their co-occurrence on the individual’s phenotype and functional outcome. Research comparing adults with ASD and SPD, as well as the impact of their co-occurrence on outcomes is extremely limited. We investigated executive functioning in terms of response inhibition and sustained attention, candidate endophenotypes of both conditions, in adults with ASD, SPD, comorbid ASD and SPD, and neurotypical adults using both categorical and dimensional approaches.

**Methods:**

A total of 88 adults (Mean Age = 37.54; SD = 10.17): ASD (*n* = 26; M/F = 20/6); SPD (*n* = 20; M/F = 14/6); comorbid ASD and SPD (*n*=9; M/F=6/3) and neurotypicals (*n*=33; M/F=23/10) completed the Sustained Attention to Response Task (SART) in both its fixed and random forms. Positive and autistic symptom severity was assessed with the positive subscale of the Positive and Negative Syndrome Scale (PANSSpos) and the PANSS Autism Severity Score (PAUSS), respectively.

**Results:**

Controlling for full scale IQ, working memory and medication dosage, group analyses revealed that the comorbid group committed fewer omission errors than the ASD group on the fixed SART, and fewer omission errors than the ASD and SPD groups on the random SART. The individual difference analyses of the entire sample revealed that the PANSSpos and PAUSS interactively reduced omission errors in both the fixed and random SARTs, as well as increased d’ scores, indicative of improved overall performance. We observed no significant results for commission errors or reaction time.

**Conclusions:**

Concurrent elevated levels of autistic and positive psychotic symptoms seem to be associated with improved sustained attention abilities (reduced omission errors) but not inhibition (commission errors). Our findings highlight the importance of investigating the concurrent effect of ASD and SPD at both the symptom and diagnostic levels, and raise important questions for future research regarding the clinical and behavioral phenotypes of adults with dual diagnosis and, more generally, about the nature of the relationship between ASD and SPD.

## Introduction

Autism spectrum disorder (ASD) and schizotypal personality disorder (SPD) are considered diagnostically independent (1). ASD is a neurodevelopmental disorder typically associated with impairments in social development, language, and repetitive, circumscribed behaviours/interests. SPD is a nonpsychotic schizophrenia spectrum disorder (SSD) involving milder symptoms of schizophrenia, and can be diagnosed in children as young as 6 years of age (2–4). However, the nosologic separation between them is not clear (5), particularly in light of accumulating evidence suggesting that ASD and SPD share etiological and risk factors, and that they can co-occur at both the diagnostic and symptom/trait levels (6–8). For example, reports show that 41% of adolescents with ASD met the DSM-IV-TR diagnostic criteria for SPD (9). Moreover, schizotypal symptoms are found at significant levels in children with ASD (2), and vice versa (10). This raises important questions about the nature of their association and the effect of their co-occurrence on the individual’s phenotype and functional outcome. It has been recommended that informing etiological and phenotypic overlaps between ASD and SSD would require the utilization of a dual-diagnosis cohort compared with two control groups, each singly diagnosed with ASD or SSD (11), and that the development of a multidimensional model for understanding the relationship between these two spectra would require cohorts to be described not solely by diagnosis, but also by using dimensional measures that cut across diagnostic boundaries (11–14). To fill in this gap, the current study investigated executive functioning in terms of response inhibition and sustained attention in adults with ASD, SPD, comorbid ASD and SPD (CM), and neurotypical adults using both categorical and dimensional approaches.

Dysfunction associated with sustained attention and inhibition has been proposed as endophenotypes for both conditions (15–17), and thus they represent common features wherein the relationship between the two disorders can be evaluated. Since we examine sustained attention and inhibition with The Sustained Attention to Response Task (SART) (18), our survey of the literature has primarily focused on studies that have utilized this task in particular in both its random and fixed versions (see Materials and Methods). Research in SSD, both at the diagnostic and dimensional levels, reports performance difficulties on the SART (19–21). For example, O’Gráda et al. (19) showed that the schizophrenic group was more impaired than controls on sustained attention (measured through omission errors), but not inhibition (measured through commission errors), and that severity of negative symptoms correlated with difficulties in sustaining attention. Another study (20) found no statistically significant differences in commission errors between healthy controls, individuals with schizotypal features, and schizophrenic patients, nor an association between schizotypal features or schizophrenia symptoms with any of the SART’s performance indices. However, it reported differences in overall task performance, with the schizotypy group intermediately positioned. With respect to ASD, one study (22) showed that while the ASD children did not show sustained attention deficits (measured through omission errors), it showed dissociation in response inhibition performance (measured through commission errors), but only on the random version of the SART. Similar results were reported in elderly with ASD while performing the fixed SART (23); compared to controls, they made more commission errors and a similar number of omission errors. A later study (24) also reported the absence of sustained attention deficits in ASD children, but not for those with comorbid ADHD.

Research directly comparing ASD and SSD on executive function in adults is extremely limited. In one study, Demetriou et al. (25) compared executive function in young adults with ASD, Early Psychosis, and Social Anxiety Disorder, using a battery of neuropsychological and self-report assessments. Relative to the typically developing group, the ASD group was impaired on mental flexibility, sustained attention and fluency, while the early psychosis group was impaired on sustained attention and attentional shifting. Notably, the early psychosis group was significantly more impaired than the ASD group on sustained attention. To our knowledge, only one study (26)—albeit in male children—compared response inhibition in ASD and SSD using the fixed version of the SART. They found that both the ASD and SSD groups had significantly lower correct responses than the typically developing group, and that the SSD group had slower reaction time and lower efficiency than the ASD group. With respect to response inhibition, the commission error rate in the ASD group was higher than the typical developing group, and non-significantly different from the SSD group.

Taken together, results from previous SART studies in ASD and SSD suggest that while ASD appears to be primarily associated with response inhibition problems, SSD appears to be associated with sustained attention deficits.

We are only aware of one study, performed in children, that has directly compared executive functioning in ASD, SPD, and CM groups (3). Results showed that while the overall performance of the ASD and SPD groups on the intra-/extra-dimensional set-shifting (IED) task was worse than the typically developing group, the overall performance of the CM group was significantly better than the ASD and SPD groups, and not significantly different from the typically developing group. Interestingly, relative to the typically developing group, clear distinctions between the ASD and SPD groups were present. Specifically, the ASD group had difficulties with extra-dimensional shifts, and the SPD group with intra-dimensional shifts. The study found no differences between the groups in non-verbal short-term or working memory, or response inhibition.

Given previous findings from studies using the SART, it was hypothesized that the frank clinical groups would demonstrate performance deficits on the SART relative to the neurotypical group. Specifically, relative to the neurotypical group, we predicted worse performance on response inhibition for the ASD group, and worse performance on sustained attention for the SPD group. In addition, based on evidence for improved performance in children with comorbid ASD and SPD on the IED task (3), and the fact that performance on the SART requires the recruitment of both sustained attention and response inhibition (22), we hypothesized that the CM group might perform better than the ASD and SPD groups, and that it would show no or attenuated impairment relative to the neurotypical group. This hypothesis is conceivable if we assume that response inhibition and sustained attention represent two poles of irregularities across the autism and schizotypal spectra that converge in a compensatory manner in the CM group. From a dimensional perspective, a corollary hypothesis would be to expect performance benefits in individuals jointly expressing elevated levels of autistic and positive psychotic symptoms.

## Materials and Methods

### Participants

A total of 88 individuals participated in the study (Mean age (SD) = 37.54(10.17); Male/Female = 63/25). The sample, which has been previously used in another study (27), consisted of an ASD, SPD, comorbid (CM), and neurotypical (NT) control groups (see **Table 1** for demographic and clinical details). As previously described (27), individuals with ASD were recruited from clinical and support services in Southeast Scotland. All had a DSM-IV diagnosis of either autism or Asperger Syndrome and met ASD cut-offs on the Autism Diagnostic Observational Schedule-Generic (ADOS-G) (28). Individuals with SPD were recruited from nonpsychotic people who had previously participated in the Edinburgh High Risk Study of schizophrenia (EHRS) (29), and from clinical services in Southeast Scotland. All met DSM-IV criteria for SPD using the Structured Clinical Interview for DSM-IV Axis II Disorders (SCID-II) (30). Individuals in the comorbid group met criteria for both ASD (determined by DSM-IV and the ADOS) and SPD (determined by the SCID-II). Finally, controls were recruited from participant and investigator acquaintances and the Scottish Mental Health Network research register. Individuals with a history of, or first degree relative with ASD, SPD, or a psychotic illness were excluded. General exclusion criteria were IQ < 70, substance dependence or history of schizophreniform disorder, schizophrenia or bipolar affective disorder. Full Scale Intelligence Quotients (FSIQ) was assessed with the Wechsler Abbreviated Intelligence Scale (31). The study was approved by the NHS Lothian Research Ethics Committee. Written informed consent was obtained from all participants.

**Table 1:** Demographics and clinical characteristics of the study groups.

Variables*	NT (N = 33)	ASD (N = 26)	SPD (N = 20)	CM (N = 9)	Stat F/χ^2^/H	p-value
Gender (M:F)	23:10	20:6	14:6	6:3	0.72^b^	0.88
Age	36.53 (9.33)	39.65 (11.89)	37.26 (9.42)	35.80 (10.03)	0.56^a^	0.64
FSIQ	118.06 (9.86)	114.81 (16.75)	106.40 (10.69)	102.44 (23.61)	4.68^a^	**0.005** SPD, CM < NT^d^
LNS Level	5.64 (1.19)	4.62 (1.13)	5.00 (1.38)	3.89 (1.54)	14.35^c^	**0.002** ASD, CM < NT^d^
LNS Total	13.39 (2.65)	11.54 (3.09)	11.60 (3.47)	10.7 (4.30)	7.67^c^	0.053
PANSS positive	7.52 (1.16)	9.92 (2.67)	12.95 (2.37)	14.11 (2.42)	48.05^c^	**<0.001** SPD, CM > ASD > NT^d^
PAUSS	8.00 (0.00)	12.88 (4.29)	11.63 (3.18)	14.89 (5.21)	33.12^c^	**<0.001** ASD, SPD, CM > NT^d^
PAUSS Social	3.00 (0.00)	4.66 (2.21)	4.47 (2.09)	5.44 (2.24)	19.11^c^	**<0.001** ASD, SPD, CM > NT^d^
PAUSS Communication	2.00 (0.00)	3.46 (1.77)	3.00 (1.25)	4.67 (2.29)	22.90^c^	**<0.001** ASD, SPD, CM > NT^d^
PAUSS Stereotypies	3.00 (0.00)	4.77 (1.53)	4.16 (1.50)	4.78 (1.64)	18.48^c^	**<0.001** ASD, CM > NT^d^
CPZ	0.00 (0.00)	3.85 (13.56)	23.75 (52.24)	63.89 (135.27)	12.73^c^	**0.005** SPD, CM > NT^d^

*Continuous variables are presented in means with standard deviations.

M, Male; F, Female; FSIQ, Full Scale Intelligence Quotients; LNS, Letter Number Sequencing; PANSS positive, Positive Subscale of the Positive and Negative Syndrome Scale; PAUSS, PANSS Autism Severity Scale; CPZ, Chlorpromazine equivalents;

^a^F statistics; ^b^Fisher’s exact test; ^c^Kruskal-Wallis Test (H); ^d^Bonferroni corrected. The p-values are indicated under the p-value column (right most column), and significant values are in bold.

### Assessments

In addition to the ADOS-G, the SCID-II, and FSIQ, all participants were assessed with the Positive and Negative Syndrome Scale (PANSS) (32). From the PANSS, both the positive and the PANSS autism severity score (PAUSS) subscale were calculated. The PAUSS (33) is a validated dimensional measure of autism symptom severity in individuals with schizophrenia, and consists of PANSS items indicative of autistic behavior: difficulties in social interaction (Items N1, N3, N4), difficulties in communication (Items N5, N6), and limited, repetitive, and stereotypic patterns of behavior (Items N7, G5, G15). The PAUSS has been shown to be a sensitive measure of autism symptom severity in young people with first-episode psychosis (34), and in individuals with schizophrenia (35–38). The internal consistency of the PAUSS in this study was fair (Cronbach’s α = 0.75). For the PANSS positive, Cronbach’s α was 0.62. However, the average inter-item correlation was good (r_IICorAvg_ = 0.164), which is a more suitable measure of internal consistency for scales less than 10 items (39).

For those on antipsychotic medication, doses were converted to chlorpromazine (CPZ) equivalents (40, 41).

### Working Memory

Following O’Gráda et al. (19), we included working memory to index higher ‘executive’ functioning, which was assessed using the letter-number sequencing (LNS) task from the Wechsler Adult Intelligence Scale, 3rd edition [WAIS-III, (42)]. In this task, individuals were presented with a pseudorandom series of numbers and letters. They were then asked to respond with the numbers first in numerical order, followed by the letters in alphabetical order. The task consisted of 7 levels with gradually increasing number of components (ranging from level 1 with two components – one letter and one number, to level 7 with 8 components). Each level contained 3 items. For the current study, performance on the LNS was considered for the level reached and the total number of correctly recalled sequences (Maximum score = 21).

### The Sustained Attention Response to Task (SART)

**Figure 1:**
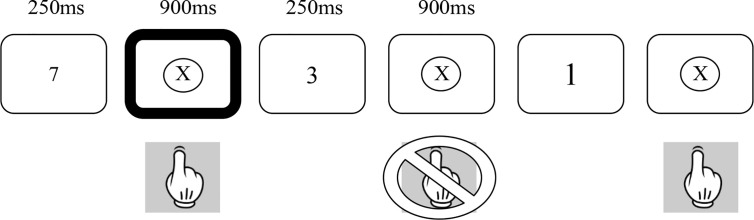
Schematic diagram of the Random Sustained Attention to Response Task.

The SART (18) was employed in both its fixed and random forms (22). **Figure 1** provides a summary of the random version of the task. In both forms, numbers between 1 and 9 were presented on a laptop screen 225 times over 4 min and 19 s. The numbers were in one of 5 different font sizes and no font size occurred more than twice in a row. Each number appeared on the screen for 250 ms and was followed by a mask (a cross in a circle) for 900 ms. Participants were asked to press the space bar for every number (Go trials) except for the number 3 (No-go trials). In order to minimize impulsive responses, they were asked to not press the space bar until the appearance of the mask. In the fixed form of the SART, the numbers are presented in repeated cycles of a fixed ascending order (i.e., 1, 2, 3, 5, 6, 7, 8, 9, 1, 2,…). In the random form, the numbers are presented in a pseudorandom order. In both versions, each number appears 25 times. All participants completed the Fixed SART followed by the Random SART.

The SART differs from traditional continuous performance tasks in that it requires the inhibition of response to an infrequent target as opposed to requiring a response to an infrequent target. Withholding of the primed response is suggested to place greater load on sustained attention networks (18). Clearly, in addition to sustained attention, individuals must also show intact response inhibition to perform the SART. To the extent that response inhibition and sustained attention can be dissociable, the use of the fixed and random forms of the SART allows these two aspects of performance to be dissociated. The Random SART places greater load on inhibitory functions than the Fixed SART due to the random presentation of either Go or No-go trials, whereas the Fixed SART places relatively greater demand on attentional compared to inhibitory functions due to the predictable nature of the Go and No-go trials (22).

Performance on the SART is measured through the number of omission (failed Go trials) and commission (failed No-go trials) errors. Omission errors on both versions of the SART are related to lapses in sustained attention. Commission errors on the random SART are related to difficulties in both sustained attention and response inhibition, whereas commission errors on the Fixed SART are primarily related to lapses in sustained attention with a much smaller load being placed upon response inhibition. In addition, overall performance, d-prime (d’), was calculated as the standardized difference between hits and false alarms as follows: d’ = z(H) - z(F). A correction was applied when the rate of false alarms was zero [1/(2N_lures_)], and when the rate of hits was one [1-1/(2N_targets_)].

We also recorded response reaction time (RT) of correct responses for both tasks.

### Statistical Analyses

Differences in demographics and clinical variables of the groups were analyzed using F, χ^2^, and H statistics, as appropriate. Group analyses of the omission and commission errors of the fixed and random SART tasks were performed using Generalized Linear Models (GLMs) with negative binomial distribution, using Wald chi-square statistics. A negative binomial distribution is appropriate for the analysis of count data and when the expected variance is greater than the mean (43, 44). The shape parameter *k* of the negative binomial distribution of each of the omission/commission errors was calculated as follows: $k=\frac{m^{2}}{v-m},$ where *m* is the mean and *v* is the variance (43). *d’* scores, indicative of overall performance on the SART tasks, were analyzed with GLMs, using the *identity* link function. Mean reaction time to correct responses was analyzed with GLMs, using the *log* link function. All group analyses were conducted while controlling for FSIQ, LNS level, and CPZ on which the groups differed (see **Table 1**).

Individual difference analyses of SART outcome measures were also analyzed as a function of PANSSpos, PAUSS and their interaction using GLMs as above, while controlling for FSIQ, LNS level, CPZ, and diagnosis. Analyses were performed using SPSS Version 24. Significant interactions were probed with the Johnson-Neyman method in R Studio (45). The Johnson-Neyman method provides a “high-resolution picture” of the interaction by estimating the value(s) of one predictor at which the other predictor has a significant effect on the outcome measure. This is established by identifying the precise value(s) along the continuum of one predictor for which the regression slopes of the other predictor are estimated to be significantly different from zero.

Unless it is otherwise noted, all p-values are FDR adjusted (*q*-value = 0.05) for multiple testing (46). Effect sizes are reported in terms of Pseudo-R^2^ and Cohen’s d.

## Results

Preliminary analyses are presented in **Tables 1** and **2**. **Table 1** presents the demographics and clinical characteristics of the study groups. Group comparisons did not reveal differences in age, gender distribution, or the total number of correctly recalled sequences of the LNS task. However, significant group differences were observed in FSIQ, LNS level, and CPZ dosage.

**Table 2** presents the correlations between the study variables. We note that neither the PAUSS nor the PANSS positive significantly correlated with either the Fixed or Random SART outcome measures.

**Table 2:** Spearman’s correlations between the study variables in the entire sample*.

Variable	1	2	3	4	5	6	7	8	9	10	11	12	13	14	15
1. Age		0.02	-0.02	-0.07	-0.04	0.18	0.22	-0.09	0.00	-0.02	-0.08	0.05	0.08	0.02	0.03
2. FSIQ	0.02		**0.42**	**0.47**	-0.18	-0.25	**-0.29**	**-0.42**	**-0.36**	**-0.28**	**-0.29**	**0.39**	**0.34**	-0.10	-0.01
3. LNS Level	-0.01	**0.42**		**0.89**	**-0.30**	-0.18	**-0.35**	**-0.32**	**-0.38**	**-0.29**	-0.23	**0.37**	**0.29**	-0.19	-0.06
4. LNS Total	-0.07	**0.47**	**0.89**		-0.18	-0.11	-0.24	**-0.35**	**-0.42**	**-0.28**	**-0.30**	**0.42**	**0.33**	-0.13	0.03
5. CPZeq	-0.04	-0.18	**-0.30**	-0.18		**0.41**	0.24	0.08	0.13	0.05	-0.07	-0.11	0.02	**0.25**	0.20
6. PANSS Pos.	0.18	**-0.25**	-0.18	-0.11	**0.41**		**0.51**	0.15	0.09	0.01	0.08	-0.14	-0.08	0.05	0.05
7. PAUSS	0.22	**-0.29**	**-0.35**	**-0.24**	0.24	**0.51**		0.17	0.08	0.02	0.05	-0.14	-0.07	0.13	0.08
8. F SART OE	-0.09	**-0.42**	**-0.32**	**-0.35**	0.08	0.15	0.17		**0.66**	**0.56**	**0.60**	**-0.89**	**-0.68**	-0.21	**-0.26**
9. F SART CE	0.00	**-0.36**	**-0.38**	**-0.42**	0.13	0.09	0.08	**0.66**		**0.47**	**0.58**	**-0.91**	**-0.62**	-0.11	**-0.26**
10. R SART OE	-0.02	**-0.28**	**-0.29**	**-0.28**	0.05	0.01	0.03	**0.56**	**0.47**		**0.47**	**-0.56**	**-0.68**	-0.04	-0.06
11. R SART CE	-0.08	**-0.29**	**-0.23**	**-0.30**	-0.07	0.08	0.05	**0.60**	**0.58**	**0.47**		**-0.66**	**-0.95**	**-0.50**	**-0.71**
12. F SART d’	0.05	**0.39**	**0.37**	**0.42**	-0.11	-0.14	-0.14	**-0.89**	**-0.91**	**-0.56**	**-0.66**		**0.72**	0.19	**0.31**
13. R SART d’	0.08	**0.34**	**0.29**	**0.33**	0.02	-0.08	-0.07	**-0.68**	**-0.62**	**-0.68**	**-0.95**	**0.72**		**0.42**	**0.58**
14. F CR RT	0.02	-0.10	-0.19	-0.13	**0.25**	0.04	0.13	-0.21	-0.11	-0.04	**-0.50**	0.19	**0.42**		**0.66**
15. R CR RT	0.03	-0.01	-0.06	0.03	0.20	0.05	0.08	**-0.26**	**-0.26**	-0.06	**-0.71**	**0.31**	**0.58**	**0.66**	

FSIQ, Full Scale Intelligence Quotients; LNS, Letter Number Sequencing; PANSS pos, Positive Subscale of the Positive and Negative Syndrome Scale; PAUSS, PANSS Autism Severity Scale; CPZ, Chlorpromazine equivalents; F SART, Fixed SART; R SART, Random SART; OE, Omission errors; CE, Commission errors; d’, d prime; F CR RT, Fixed SART Correct Responses Mean Reaction Time; R CR RT, Random SART Correct Responses Mean Reaction Time;

* Coefficients in bold are significant (p < 0.05). Coefficients above the diagonal are FDR adjusted for multiple tests.

### Group Differences in Fixed and Random SART

**Figure 2:**
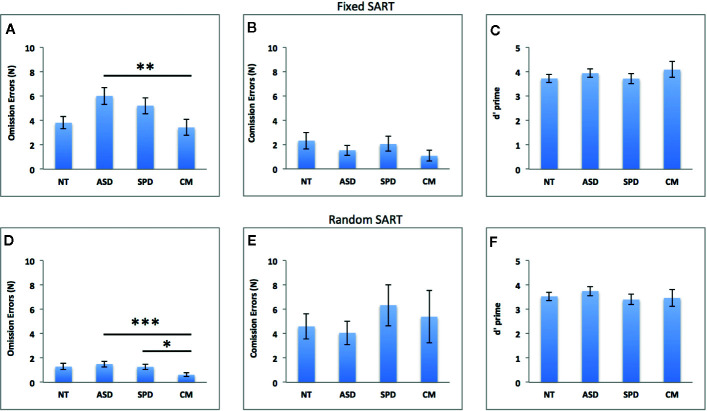
Groups comparisons on omission errors, commission errors and overall performance (d’) in the fixed **(A–C)** and random **(D–F)** SART tasks. NT, Neurotypical Controls; ASD, Autism Spectrum Disorder; SPD, Schizotypal Personality Disorder; CM, Comorbid group. Error bars represent standard error of the mean (SEM). *p < 0.05, **p < 0.01, ***p < 0.001.

**Figure 3:**
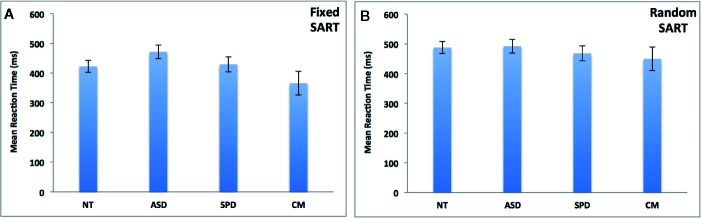
Groups comparisons on mean response time (in milliseconds) of appropriate responses in the fixed **(A)** and random **(B)** SART tasks. NT, Neurotypical Controls; ASD, Autism Spectrum Disorder; SPD, Schizotypal Personality Disorder; CM, Comorbid group. Error bars represent standard error of the mean (SEM).

**Figure 2** depicts the results of the group analyses on omission and commission errors and overall performance (d’) of the fixed and random SART tasks. **Figure 3** depicts the results of the group analyses on mean reaction time of correct responses of the fixed and random SART tasks.

#### Fixed SART Omission Errors

The overall model was significant (χ^2^ = 63.71, df = 6, p*_corr_* < 0.001, Pseudo R^2^ = 0.12). As can be seen in **Figure 2A**, there was a significant main effect for group (Waldχ^2^ = 13.94, df = 3; p = 0.003) such that the CM group made fewer errors than the ASD group (MD(se) = -2.59(0.77), *p_cor_*_r_ = 0.005, Cohen’s *d* = 0.79). The ASD group made more errors than the NT group at a trend level (MD(se) = 2.20(0.89); *p_cor_*_r_ = 0.065, Cohen’s *d* = 0.68). This was independent of the significant effect of FSIQ, where increasing FSIQ scores were associated with fewer errors (β(se) = - 0.009(0.002), Waldχ^2^ = 33.64, df = 1; p*_corr_* < 0.001).

#### Fixed SART Commission Errors

The overall model was non-significant (χ^2 =^ 7.61, df = 6, p*_corr_* = 0.357). See **Figure 2B**.

#### Fixed SART d Prime

The overall model was significant (χ^2^ = 18.58, df = 6, p*_corr_* = 0.013, Pseudo R^2^ = 0.19). Better task performance was significantly associated with FSIQ (β(se) = 0.018(0.008), Waldχ^2^ = 5.45, df = 1; p = 0.020), and higher LNS levels (β(se) = 0.181(0.087), Waldχ^2^ = 4.30, df = 1; p = 0.038). However, as can be seen from **Figure 2C**, the difference between the groups was non-significant (Waldχ^2^ = 1.64, df = 3; p = 0.644).

#### Fixed SART Mean Reaction Time of Correct Responses

The overall model was non-significant (χ^2 =^ 9.10, df = 6, p*_corr_* = 0.269). See **Figure 3A**.

#### Random SART Omission Errors

The overall model was significant (χ^2^ = 143.55, df = 6, p*_corr_* < 0.001, Pseudo R^2^ = 0.37). There was a significant main effect of group (Waldχ^2^ = 31.72, df = 3; p < 0.001) such that the CM group made fewer errors than the ASD (MD(se) = -0.88(0.18); *p_corr_* < 0.001, Cohen’s *d* = 0.80) and SPD (MD(se) = -0.64 (0.21); p*_corr_* = 0.012, Cohen’s *d* = 0.74) groups (see **Figure 2D**). This was independent of the significant effects of FSIQ, LNS levels and CPZ dosage, where increasing FSIQ (β(se) = - 0.031(0.004), Waldχ^2^ = 66.80, df = 1; p < 0.001), and LNS level (β(se) = - 0.173(0.057), Waldχ^2^ = 9.11, df = 1; p = 0.003) were associated with fewer errors, while higher CPZ dosage was associated with more errors (β(se) = 0.003(0.001), Waldχ^2^ = 5.37, df = 1; p = 0.021).

#### Random SART Commission Errors

The overall model was non-significant (χ^2 =^ 4.91, df = 6, p*_corr_* = 0.635). See **Figure 2E**.

#### Random SART d Prime

The overall model was significant (χ^2^ = 15.24, df = 6, p*_corr_* = 0.037, Pseudo R^2^ = 0.16). Task performance was marginally associated with FSIQ (β(se) = 0.015(0.008), Waldχ^2^ = 3.48, df = 1; p = 0.062) and LNS level (β(se) = 0.161(0.093), Waldχ^2^ = 3.02, df = 1; p = 0.082). The difference between the groups was non-significant (Waldχ^2^ = 1.52, df = 3; p = 0.678). See **Figure 2F**.

#### Random SART Mean Reaction Time of Correct Responses

The overall model was non-significant (χ^2 =^ 1.83, df = 6, p*_corr_* = 0.935). See **Figure 3B**.

### Individual Difference Analyses: Fixed SART

#### Fixed SART Omission Errors

The overall model was significant (χ^2^ = 65.85, df = 9, p*_corr_* < 0.001, Pseudo R^2^ = 0.33). Parameter estimates revealed a significant negative PAUSS x PANSSp interaction on omission errors (β(se) = - 0.067(0.027), Waldχ^2^ = 6.21, df = 1; p = 0.013).

#### Fixed SART Commission Errors

The overall model was non-significant (χ^2 =^ 8.64, df = 9, p*_corr_* = 0.539).

#### Fixed SART d Prime

The overall model was significant (χ^2^ = 24.23, df = 9, p*_corr_* = 0.011, Pseudo R^2^ = 0.30). Parameter estimates revealed a significant positive PAUSS x PANSSp interaction on d’ prime (β(se) = 0.302(0.101), Waldχ^2^ = 8.94, df = 1; p = 0.003).

**Figure 4:**
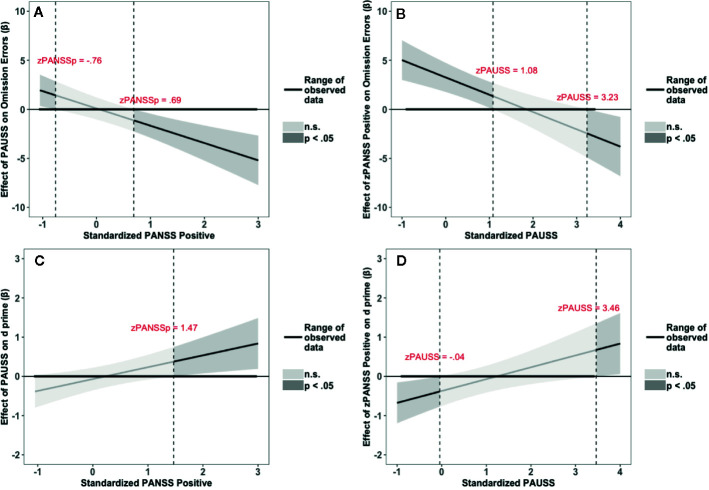
Results of the Johnson-Neyman interaction probes for omission errors (OE) and d prime scores (d’) of the Fixed Sustained Attention to Response Task (SART) task. **(A, C)** depict the association (β weights) of the Positive and Negative Syndrome Scale (PANSS) Autism Severity (PAUSS) scores with OE and d’, respectively, along the range of the standardized values of the PANSS positive scores. **(B, D)** depict the association (β weights) of the PANSS positive scores with OE and d’, respectively, along the range of the standardized values of the PAUSS scores. Areas shaded in dark grey represent the zone of significant effects (p < 0.05), and areas shaded in light gray represent the zone of non-significant effects (p > 0.05). Slopes are bounded by 95% confidence intervals.

The results of the interaction probes for the omission errors and overall performance (d’) of the fixed SART task are summarized in **Figure 4**. **Figures 4A, B** depict the results for the omission errors. **Figure 4A** shows that the PAUSS is associated with a significant increase in omission errors when PANSS positive is ≤ - 0.76 SD from the mean, but with a significant decrease in errors when PANSS positive is ≥ 0.69 SD from the mean. Conversely, **Figure 4B** shows that PANSS positive is significantly associated with an increase in errors when PAUSS is ≤ 1.08 SD from the mean, but with a significant decrease in errors when PAUSS is ≥ 3.23 SD from the mean.

**Figures 4C, D** depict the results for d’. **Figure 4C** shows that PAUSS is significantly associated with better performance when PANSS positive is ≥ 1.47 SD from the mean. Conversely, **Figure 4D** shows that PANSS positive is significantly associated with worse performance when PAUSS is ≤ - 0.04 SD from the mean, but with significantly better performance when PAUSS is ≥ 3.46 SD from the mean, albeit this is outside the range of the PAUSS scores in our data [PAUSS range = -0.89, 3.40].

#### Fixed SART Reaction Time

The overall model was non-significant (χ^2 =^ 11.71, df = 9, p*_corr_* = 0.368).

### Individual Difference Analyses: Random SART

#### Random SART Omission Errors

The overall model was significant (χ^2^ = 37.80, df = 9, p*_corr_* < 0.001, Pseudo R^2^ = 0.41). Parameter estimates revealed a significant and negative PAUSS x PANSS positive interaction on omission errors (β(se) = - 0.24(0.102), Waldχ^2^ = 5.60, df = 1; p = 0.018).

#### Random SART Commission Errors

The overall model was non-significant (χ^2 =^ 7.44, df = 9, p*_corr_* = 0.591).

#### Random SART D’ Prime

The overall model was significant (χ^2^ = 23.35, df = 9, p*_corr_* = 0.011, Pseudo R^2^ = 0.291). Parameter estimates revealed a significant and positive PAUSS x PANSS interaction on errors (β(se) = 0.253(0.110), Waldχ^2^ = 5.24, df = 1; p = 0.022).

**Figure 5:**
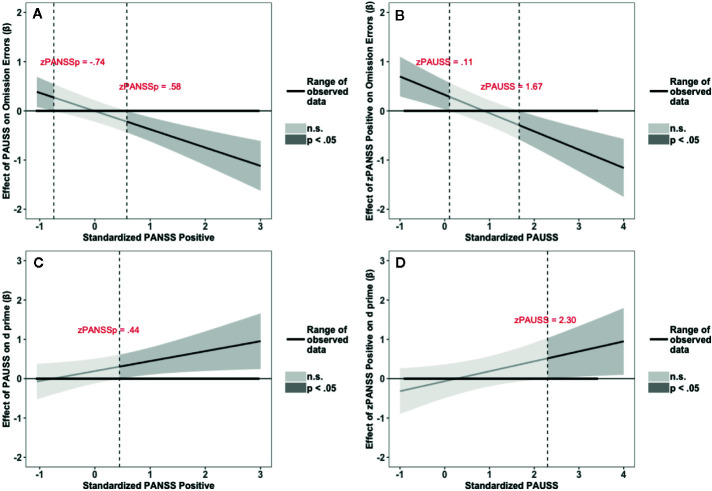
Results of the Johnson-Neyman interaction probes for omission errors (OE) and d prime scores (d’) of the Random Sustained Attention to Response Task (SART) task. **(A, C)** depict the association (β weights) of the Positive and Negative Syndrome Scale (PANSS) Autism Severity (PAUSS) scores with OE and d’, respectively, along the range of the standardized values of the PANSS positive scores. **(B, D)** depict the association (β weights) of the PANSS positive scores with OE and d’, respectively, along the range of the standardized values of the PAUSS scores. Areas shaded in dark grey represent the zone of significant effects (p < 0.05), and areas shaded in light grey represent the zone of non-significant effects (p > 0.05). Slopes are bounded by 95% confidence intervals.

The results of the interaction probes for the omission errors and overall performance (d’) of the random SART task are summarized in **Figure 5**. **Figures 5A, B** depict the results for the omission errors. **Figure 5A** shows that PAUSS is associated with an increase in omission errors when PANSS positive is ≤ - 0.74 SD from the mean, but with a significant decrease in errors when PANSS positive is ≥ 0.58 SD from the mean. Conversely, **Figure 5B** shows that PANSS positive is associated with an increase in omission errors when PAUSS is ≤ 0.11 SD from the mean, but with a significant decrease in errors when PAUSS is ≥ 1.67 SD from the mean. **Figures 5C, D** depict the results for d’. **Figure 4C** shows that PAUSS is significantly associated with better overall performance when PANSS positive is ≥ 0.44 SD from the mean. Conversely, **Figure 4D** shows that PANSS positive is significantly associated with better overall performance when PAUSS is ≥ 2.30 SD from the mean.

#### Random SART Reaction Time

The overall model was non-significant (χ^2 =^ 10.69, df = 9, p*_corr_* = 0.397).

### Exploratory Analyses

To gain further insight into the association of the interaction of PANSS positive x PAUSS scores with reduced omission errors, we performed a serious of exploratory analyses in the entire sample as well as in each of the ASD and SPD groups, separately. First, for the entire sample, we examined the association of PANSS positive with each of the three subdomains of the PAUSS (i.e., social difficulties, communication difficulties, and stereotypies/narrowed interests) with omission errors in both the fixed and random SART tasks to see if the interactions we observed in the main analyses were driven by a specific subdomain of autistic features. For each model, we examined the association of PANSS positive and its interaction with each of the PAUSS subdomains while controlling for the other two subdomains as well as for FSIQ, LNS level, CPZ, and diagnosis. In the fixed SART, omission errors were associated with a negative PANSS positive x PAUSS stereotypic behavior interaction (β(se) = -0.112(0.037), Waldχ^2^ = 9.11, df = 1; p = 0.003). The interactions of PANSS positive with the PAUSS social (p = 0.131) and PAUSS communication (p = 0.955) subdomains were non-significant. For the Random SART, omission errors were associated with a negative PANSS positive x PAUSS stereotypic behavior interaction (β(se) = -0.547(0.133), Waldχ^2^ = 16.94, df = 1; p < 0.001), as well as with a negative PANSS positive x PAUSS communication interaction (β(se) = -0.323(0.129), Waldχ^2^ = 6.24, df = 1; p = 0.013). The interaction of PANSS positive with the PAUSS social subdomain was non-significant (p = 0.076).

**Figure 6:**
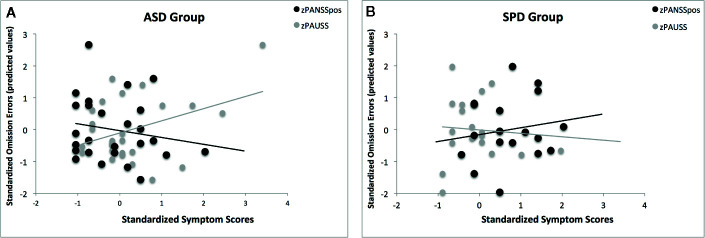
The association of the PANSS Autism Severity (PAUSS) and PANSS Positive (PANSSpos) scores with omission errors on the Random SART task in the ASD and SPD groups. **(A)** shows the association of the standardized PAUSS and PANSSpos scores with the standardized predicted values of the omission errors in the ASD group, where PAUSS scores are associated with increased omission errors and the PANSSpos scores are associated with reduced omission errors. Here, the interaction of PAUSS x PANSSpos is significant ASD (β(se) = -0.736(0.318), Waldχ^2^ = 5.37, df = 1; p = 0.021)**. (B)** shows the association of the standardized PAUSS and PANSSpos scores with the standardized predicted values of the omission errors in the SPD group, where PANSSpos scores are associated with increased omission errors (β(se) = 2.595(0.731), Waldχ^2^ = 12.60, df = 1; p < 0.001) and the PAUSS scores are associated with reduced omission errors (β(se) = -2.736(0.976), Waldχ^2^ = 8.63, df = 1; p = 0.003). Here, the PAUSS x PANSSpos interaction is not significant (p = 0.281).

Following the same analyses we performed for the entire sample, we explored the association of the PANSS positive x PAUSS interaction with omission errors in the ASD only group, and in the SPD only group. The results revealed significant models only for the random SART in both the ASD (χ^2^ = 17.10, df = 6, p*_corr_* = 0.018, Pseudo R^2^ = 0.52) and SPD (χ^2^ = 21.49, df = 6, p*_corr_* = 0.004, Pseudo R^2^ = 0.70) groups. In the ASD group, the PANSS positive x PAUSS interaction was associated with reduced omission errors (β(se) = -0.736(0.318), Waldχ^2^ = 5.37, df = 1; p = 0.021). In the SPD group, while the interaction was not significant (p = 0.281), the main effects of the PANSS positive and PAUSS were significant, such that increasing PANSS positive scores were associated with increased omission errors (β(se) = 2.595(0.731), Waldχ^2^ = 12.60, df = 1; p < 0.001), and increasing PAUSS scores were associated with reduced omission errors (β(se) = -2.736(0.976), Waldχ^2^ = 8.63, df = 1; p = 0.003). As can be seen from **Figures 6A, B**, the pattern of associations of PAUSS and PANSS positive with omission errors in the ASD group was reversed in the SPD group.

## Discussion

Using the SART task, we examined executive functioning in terms of response inhibition and sustained attention, two candidate endophenotypes in both ASD and SPD. Overall, we found that while the clinical groups did not differ from healthy controls, there were clear differences between the single diagnosis groups and the CM group in sustained attention (as measured with omission errors) but not response inhibition (as measured with commission errors). The group analyses revealed that the CM group committed fewer omission errors than the ASD group in both the fixed and random SART, as well as fewer errors than the SPD group in the random SART. The individual difference analyses confirmed and extended these results to show that autism and positive symptom severity interactively reduced omission errors. In addition, the individual difference analyses also revealed that the interaction was associated with better overall performance (as indexed by higher d’ values). The individual difference analyses suggest that dimensional measures are more sensitive than group level analyses, and that performance might be more aptly characterized by examining the relative severity of autistic and positive symptoms in the individual rather than the absence or presence of an ASD or SPD.

Our predictions of increased omission errors in the SPD group, and increased commission errors in the ASD group relative to the neurotypical group were not supported by our findings in either the fixed or random version of the SART. We also found no statistically significant differences between the four groups on commission errors, nor between the ASD, SPD, and NT groups on omission errors. While the lack of differences may be due to the SART being relatively an easy task to perform, these results partially overlap with findings from previous research, although caution is warranted since we are comparing our results to findings from populations with different diagnoses (schizophrenia) and at different developmental stages (children, elderly). With respect to commission errors on the fixed SART, O’Gráda et al. (19), Chan et al. (20), and Ho et al. (21) found no differences between healthy controls and schizophrenic patients, Shi et al. (26) found no differences between ASD and SSD children, and Johnson et al. (22) found no differences between ASD and typically developing children. In contrast, however, Johnson et al. (22) reported higher number of commission errors in ASD relative to typically children while performing the random SART, and Geurts et al. (23) reported similar results in elderly with ASD while performing the fixed SART. Moreover, unlike our results, O’Gráda et al. (19) found that the schizophrenic group made more omission errors than the controls while performing the fixed SART.

However, we observed differences between the clinical groups on sustained attention, with the CM group out-performing the ASD group in the fixed SART and both the ASD and SPD groups in the random SART. Cognizant of the different tasks and methodologies employed in other studies, these results are consistent with the few available studies that compared individuals with comorbid ASD-SSD to individuals with ASD or SSD. In children, the performance of those with a dual diagnosis of ASD and SPD were similar to typically developing children, and largely better than the children with the frank conditions on both attentional set shifting and socio-pragmatic skills (3). In adults, brain activations in the ASD-SPD comorbid group during a social judgment task were generally indistinguishable from the typically developing group and fell intermediately between the ASD and SPD groups (27). More recently, Sunwoo et al. (47) reported that young people with comorbid first episode psychosis (FEP) and ASD were: (1) less likely than young people with FEP only to have comorbid substance use issues, (2) more likely to be engaged in employment or education at the time of discharge, but also (3) more likely to experience impairments in interpersonal skills.

From a dimensional perspective, the results regarding the association of the interaction of PANSS autistic and positive symptoms with performance benefits on the SART resonate with those obtained for social cognition and functioning in patients with schizophrenia (38) and bipolar I disorder (48). This tentatively suggests that benefits can be observed in both the social and attentional domains in comorbid individuals at both the diagnostic and symptom level. We note, however, no such benefit was observed for social cognition and functioning in a sample of individuals with various psychotic disorders that self-reported autistic traits and positive psychotic experiences (49).

Moreover, O’Gráda et al. (19) found that severity of negative symptoms correlated with difficulties in sustaining attention. Intriguingly, this effect was reported for patients in whom the positive symptoms were low (Mean= 2.02; SD= 2.25). This appears to parallel our finding where the PAUSS scores were associated with more omission errors when positive symptoms were low (see **Figures 4A** and **5A**).

What might explain the benefit we observed in the CM group and in individual with elevated autistic and positive symptoms? As stated above, performance on the SART requires the recruitment of both sustained attention and response inhibition. However, the hypothesized dissociation between SPD and ASD in terms of these respective abilities was not supported by our results, and as such our pattern of results do not support the hypothesis that these two abilities converge in a compensatory manner in the CM group. Perhaps this is inherent in the inability of the SART task to truly dissociate inhibition from sustained attention. In this regard, Robertson et al. (18) point out that “arbitrating between the relative contributions of an inefficiency in response inhibition *per se* and a failure to inhibit responses due to a lack of continuous attention to response is of course difficult and indeed somewhat circular within this task” (p. 749).

However, the exploratory analyses provide some important leads that might be leveraged in future research to understand the mechanisms underlying benefits conferred by the co-presence of autistic and positive symptoms. First, the analyses pertaining to the subdomains of the PAUSS suggest that autistic features associated with stereotypies and narrowed interests appear to largely drive the interaction of the PAUSS total scores with PANSS positive symptoms on omission errors. This dovetails with the findings of a study on probabilistic reasoning showing that relative to individuals with delusional disorder (DD) only and individuals with obsessive-compulsive disorder (OCD) only—who respectively were reliant on less and more evidence to make their decision—probabilistic reasoning was normalized in individuals with comorbid DD and OCD (50). Stereotypic behavior is a main feature that is common to both OCD and ASD (51), and so it might be of particular importance to understanding how autistic and positive symptoms become adaptive when co-present.

Moreover, the independent analyses in the ASD and SPD groups show that the pattern of associations of PAUSS and PANSS positive symptoms with omission errors in the ASD group is reversed in the SPD (see **Figures 6A, B**). This suggests that the PAUSS and PANSS positive symptoms are associated with diametric influences on sustained attention independent of the disorder. This is consistent with (i) the diametric model (52, 53)—which posits that ASD and SSD are characterized by opposing phenotypic patterns—, (ii) evidence suggesting that ASD and SSD can be characterized by opposing patterns of attentional abilities (3, 54), and (iii) existing evidence suggesting that the presence of both disorders may be associated with attenuated impairments (3, 27, 38). Importantly, this pattern of association also suggests that the omission errors in ASD and SPD might be precipitated by different mechanisms, which is consistent with the notion that apparent overlaps between autism and schizophrenia spectrum disorders might be precipitated by different cognitive styles or biases (55, 56). Altogether, this pattern of association gives credence to the idea and that some compensatory mechanism might nonetheless be at play in the comorbid group. If so, future research (behavioral, cognitive, and neural) is necessary in order to test the prediction that these mechanisms are highly interactive and possibly of contrasting nature. Hence, assessments that require the recruitment of dissociable contrasting abilities, such as global-local processing (57, 58) and zoom-in and zoom-out attentional mechanism (59) might be particularly beneficial in discriminating between the groups and thus potentially mechanistically more informative. Within the neural domain, future research might consider the default mode and task-positive networks in search for a potential mechanism. Lapses in attention have been associated with reduced task-induced deactivation of the default mode network (60) and its anticorrelation with the task-positive network has been related to consistent behavioral performance (61). Examining disorder-specific resting state activity of these networks in ASD and SPD might provide a mechanistic account of how autism and positive symptom severity converge adaptively in sustaining attention.

We acknowledge a number of limitations of our study. First, findings of our study may be limited by the small sample size of the CM group. Thus, future work with a larger sample of CM individuals is needed in order to have a better understanding of their clinical phenotypes. Second, controls were mainly recruited through acquaintances. This recruitment strategy may have biased our sample. Third, as pointed above, the SART offered limited insight into the mechanisms that might explain the performance benefits we observed in the comorbid group. Therefore, it would be profitable for future research to implement a Research Domain Criteria (RDoC) strategy (62) for a more comprehensive assessment of the participants’ clinical and functional phenotypes that may help interpret the current results.

Fourth, while the PAUSS allows for a dimensional cross-disorder analysis (33), it has been validated against the ADOS that measures current autistic traits. As such, it may be argued, and particularly for the SPD group, that the PAUSS merely reflects the severity of later-onset, autistic-like symptoms rather than actual childhood-onset autistic traits. While, to our knowledge, the PAUSS is yet to be validated against instruments that assess childhood-onset autistic traits, nascent results suggest that PAUSS levels in schizophrenia patients with ASD, assessed with the Autism Diagnostic Interview-Revised [ADI-R, (63)]—a measure that is based on the patients’ early developmental history through a parent/caregiver interview—are similar to those of schizophrenia patients with ASD, assessed with the ADOS (35). In addition, negative symptoms in schizophrenia spectrum disorders (from which the PAUSS is largely derived) have been suggested to be of neurodevelopmental origin and predate the onset of the disorder (64, 65). Taken together, the PAUSS may be capturing childhood-onset autistic traits rather than current autistic-like features. Yet, the current lack of a unified classification instrument for cross-disorder analysis represents a general challenge to this young field, and so the development and refinement of such instruments is crucial to advance research into underlying cross-disorder mechanisms.

In conclusion, comorbid ASD-SPD or concurrent elevated levels of autistic and positive psychotic symptoms counterintuitively appear to confer greater functional advantages than simply having an ASD or SPD alone. These findings raise intriguing questions about possible mechanisms underlying the observed performance benefits. While we found no direct support for the hypothesis that sustaining attention and response inhibition converge in a compensatory manner in the comorbid group, our findings suggest that autistic and positive symptoms exert diametric influences on sustained attention abilities. More broadly, our findings highlight the importance of investigating the concurrent effect of ASD and SPD at both the symptom and diagnostic levels, and it raises important questions and directions for future research regarding the clinical and behavioral phenotypes of adults with dual diagnosis.

## Data Availability Statement

The raw data supporting the conclusions of this article will be made available by the authors, without undue reservation.

## Ethics Statement

The studies involving human participants were reviewed and approved by the NHS Lothian Research Ethics Committee. The patients/participants provided their written informed consent to participate in this study.

## Author Contributions

AS, SL, EJ, and RP designed the study and wrote the protocol. AA-A conducted statistical analysis and wrote the manuscript. All authors contributed to the article and approved the submitted version.

## Conflict of Interest

The authors declare that the research was conducted in the absence of any commercial or financial relationships that could be construed as a potential conflict of interest.
